# Role of NuMA1 in breast cancer stem cells with implications for combination therapy of PIM1 and autophagy inhibition in triple negative breast cancer

**DOI:** 10.21203/rs.3.rs-3953289/v1

**Published:** 2024-04-01

**Authors:** Kanakaraju Manupati, Mingang Hao, Michael Haas, Syn Kok Yeo, Jun-Lin Guan

**Affiliations:** Department of Cancer Biology, University of Cincinnati College of Medicine, Cincinnati, OH 45267

**Keywords:** TNBC, Autophagy, Breast cancer stem cells, NuMA1, PIM1, Combination therapy

## Abstract

**Background:**

Nuclear mitotic apparatus protein 1 (NuMA1) is a cell cycle protein and upregulated in breast cancer. However, the role of NuMA1 in TNBC and its regulation in heterogenous populations remains elusive.

**Methods:**

We performed CRISPR mediated deletion of NuMA1 in mouse TNBC cells, BF3M. FACS was utilized to isolate BCSCs, and bulk cells based on CD29 and CD61 markers. Cell viability, migration, and invasion ability of BCSCs and bulk cells was evaluated using MTT, wound healing and transwell invasion assays, respectively. In vivo mouse breast cancer and lung metastatic models were generated to evaluate the combination treatment of SMI-4a and Lys-o5 inhibitors.

**Results:**

We identified that high expression of NuMA1 associated with poor survival of breast cancer patients. Further, human tissue microarray results depicted high expression of NuMA1 in TNBC relative to non-adjacent normal tissues. Therefore, we performed CRISPR mediated deletion of NuMA1 in a mouse mammary tumor cell line, BF3M and revealed that NuMA1 deletion reduced mammary tumorigenesis. We also showed that NuMA1 deletion reduced ALDH^+^ and CD29^hi^CD61^+^ breast cancer stem cells (BCSCs), indicating a role of NuMA1 in BCSCs. Further, sorted and characterized BCSCs from BF3M depicted reduced metastasis with NuMA1 KO cells. Moreover, we found that PIM1, an upstream kinase of NuMA1 plays a preferential role in maintenance of BCSCs associated phenotypes, but not in bulk cells. In contrast, PIM1 kinase inhibition in bulk cells depicted increased autophagy (FIP200). Therefore, we applied a combination treatment strategy of PIM1 and autophagy inhibition using SMI-4a and Lys05 respectively, showed higher efficacy against cell viability of both these populations and further reduced breast tumor formation and metastasis. Together, our study demonstrated NuMA1 as a potential therapeutic target and combination treatment using inhibitors for an upstream kinase PIM1 and autophagy inhibitors could be a potentially new therapeutic approach for TNBC.

**Conclusions:**

Our study demonstrated that combination treatment of PIM1 inhibitor and autophagy inhibitor depicted reduced mammary tumorigenesis and metastasis by targeting NuMA1 in BCSCs and bulk cells of TNBC, demonstrating this combination treatment approach could be a potentially effective therapy for TNBC patients.

## Introduction

Breast cancers exhibit a high degree of intra-tumoral heterogeneity with different molecular and functional phenotypes[1, 2]. Based on molecular profile and complexity, breast cancers can be classified into subtypes such as luminal, HER-2^+^, basal-like (TNBC) and normal-like breast cancer[3, 4]. Among these, TNBC subtype is an aggressive breast cancer with high rate of mortality in women, and the intra-tumor heterogeneity within TNBC subtype are impediments for effective treatment[4, 5]. Therefore, it is essential to identify new molecular targets for the heterogenous populations within TNBC tumors to improve the survival of breast cancer patients.

Nuclear mitotic apparatus protein 1 (NuMA1) is a large cell cycle protein with a molecular weight of 238 kDa located on chromosome 11q13[6, 7]. The involvement of NuMA1 has been known in various tissue specific cancer progressions such as ovarian cancer and breast cancer[7, 8]. NuMA1 has been known to play an important role in cell division by interacting with microtubules for the formation and maintenance of mitotic spindles[7, 9]. NuMA1 protein comprises of a globular head, tail domains and long coiled-coil domains that promote NuMA1 dimerization as well as oligomerization[6, 10]. The bipartite tail domains mediate NuMA1 interaction with the mitotic spindle and the other region is involved in accurate nuclear reformation[6, 11]. NuMA1 is a major component in the interphase and nuclear matrix, it regulates asymmetric cell divisions by maintaining spindle assembly and spindle positioning via interacting with microtubule motors[11, 12]. Recent study showed that NuMA1 is binding to an ubiquitin ligase, UBE2C by recruitment of LINC01194 to promote degradation of RYR2 which acts as a negative regulator of Wnt/β-catenin signalling and promotes malignant progression of TNBC[8]. However, studies deciphering the role of NuMA1 in heterogenous cells of TNBC subtype is lacking.

PIM1 belongs to a family of serine threonine protein kinases and there are three proto-oncoproteins isoforms of PIM family such as PIM1, PIM2 and PIM3[13]. It is majorly involved in cell proliferation and survival[14]. The expression of PIM1 is correlated with poor patient survival in many types of cancers including breast cancer[14–16]. PIM1 plays an important role in cell cycle progression and apoptosis in various cancer cells[16]. It has been shown that upregulation of PIM1 in various tissues specific cancer leads to development of chemoresistance and radio-resistance[17–19]. Therefore, it is important for targeting PIM1 with other targeted therapies for better therapeutic efficacy.

Autophagy is an evolutionarily conserved self-degradation mechanism[20]. It plays a major role in recycling of intracellular molecules and damaged organelles by subjecting them to lysosomal degradation to maintain cellular homeostasis[21]. Dysregulation of autophagy has been found in many human diseases like infectious diseases, neurodegenerative disorders, metabolic diseases and cancer[22]. Autophagy involves several sequential steps including initiation, nucleation, elongation of autophagosome membrane and autophagosome fusion with the lysosome for degradation of autophagic cargos[23]. During these steps, several autophagy related proteins (ATG) including FIP200, ATG13 and ULK1, are required for initiation of autophagy[24]. The role of autophagy in cancer is complex and context dependent, with both tumor promoting and tumor suppressing functions demonstrated[25]. Despite that, autophagy inhibitors have been shown to improve chemotherapy drug responses and can be an effective class of therapeutics in certain cancer types[26–28].

In breast cancer, the existence of a small percentage of cells with stem-like cell characteristics and high metastatic initiating properties are known as breast cancer stem cells (BCSCs) which contributes to heterogeneity of breast cancer[5]. These BCSCs are resistant to chemotherapy and radiotherapy[29]. Various specific cell surface markers have been identified to examine these populations[5, 30]. Currently, the known human BCSC surface markers CD44, CD24, CD133 and ALDH have been widely characterized for the isolation of BCSCs from various breast cancer cell lines[5, 30]. A few surface markers of BCSCs were recognised in mouse mammary tumors such as CD29, CD61 and ALDH markers[25]. The presence of therapy resistant BCSCs in mammary tumors contributes to worse prognosis in breast cancer patients by promoting metastasis to distant organs like lungs, brain, bone and liver[31]. Upon stimulation, breast cancer cells with enhanced plasticity can undergo transitions from epithelial cells to mesenchymal cells (EMT) with stem-like cell properties[5, 29]. Whereas the mesenchymal cells can revert to epithelial state (MET) upon induction with external stimuli[5]. Plasticity of breast cancer cells and BCSCs adds to the challenge of targeting these populations. Therefore, it is important to develop strategies that can target both these population of cells for better cancer therapy.

## Materials and Methods

### Mouse primary breast cancer cell lines

We utilized a mouse breast cancer cell line, BF3M (TNBC) which were originated from mouse model of Brca1^F/F^ P53^F/F^ K14-Cre[32–35]. Mice were maintained in house and handled according to local, state and federal regulations. We performed all the experimental procedures according to protocols approved by Institutional Animal Care and Use committee at University of Cincinnati (Cincinnati, OH). Primary mouse tumor cell line was cultured in DMEM/F12 growth medium supplemented with 10% FBS, 1% penicillin-streptomycin, 10 ng/mL EGF and 20 mg/mL insulin. Mycoplasma tests were carried out once in a month. Cell line was utilized for experiments after tested negative for mycoplasma contamination.

### CRISPR/Cas9-mediated knockout of NuMA1

For CRISPR mediated NuMA1 deletion, we designed and synthesized three mouse specific primers for guide RNA sequences such as 1FP: 5’ –GTTGCCCCTCTTTAGTGTCA-3’. 1RP: 5’-TGACACTAAAGAGGGGCAAC-3’. 2FP: 5’ –TCTGCATGTTGCTGACCCCG-3’. 2RP: 5’-CGGGGTCAGCAACATGCAGA-3’. 3FP: 5’ –ATCCGAGATGGAACTGGCCA-3’. 3RP: 5’-TGGCCAGTTCCATCTCGGAT-3’. Next, T4 PNK (NEB M0201S), ligation buffer was used to anneal the primers at 37 °C for 30 min and 95 °C for 5 min. Ligation of lentiCRISPRv2 plasmid and annealed oligos were performed using quick ligase enzyme (NEB M2200S) followed by plasmid amplification in Stbl3 competent cells. For generation of lentiviral particles, HEK-293T cells were transfected with control vector and lentiCRISPRv2-NuMA1sgRNA-Puro vector (Addgene, USA) in presence of pMD2.G and psPAX2. Cells were then transduced with the lentiviral particles using polybrene (5 μg/mL) followed by puromycin selection [36]. Single cells were sorted in 96-well plate using flow cytometry and expanded for screening of NuMA1 deletion using immunoblot.

### Immunoblotting

Modified RIPA lysis buffer was used to prepare cell lysates as described previously [25, 36]. Next, samples were subjected to SDS-PAGE and transferred onto nitrocellulose membrane for immunoblotting with the primary antibodies: NuMA1 antibody (Abcam, ab109262), Cleaved Caspase-3 (Cell Signaling, 9661), Bad (Cell Signaling Technology, 9292T), Vinculin (Sigma Aldrich, V4505), followed by secondary antibodies, anti-mouse IgG, HRP-linked antibody (Cell Signaling Technology, 7076) and anti-rabbit IgG, HRP-linked antibody (Cell Signaling Technology, 7074). Ki-67, PIM1 (Invitrogen, MA5–35347).

### Orthotopic mouse tumor transplants and metastatic models

BF3M cells with or without NuMA1 KO (1 × 10^6^) were prepared in 50μl of PBS and transplanted into the mammary fat pads of FVB female mice. For characterization of BCSCs and bulk cells, equal number of cells (1 × 10^6^) were injected orthotopically into the fat pads of FVB female mice. After palpable tumor formation, drug treatments of Lys-05 (40mg/kg) (Selleck Chem, S8369), SMI-4a (10mg/kg) (Selleck Chem, S8005) were initiated on day1 and continued for 7 days. Tumor volume was calculated as (1/2) (length) (width)^2^ from measured tumor growths at regular intervals using calipers. Athymic nude mice was used for the generation of breast cancer metastatic model. Briefly, cells (5 × 10^5^) were injected via tail-vein of mice and monitored for 2 months for the formation of metastatic lung nodules as described previously [36].

### Immunohistochemistry

Tumors were harvested at the end point for histology. Formalin-fixed paraffin-embedded tumors were subjected to tissue sections (5 μm) followed by staining with Ki67 antibody (eBioscience, 50-245-564) and hematoxylin and eosin as described previously [36].

### Flow cytometry and isolation of BCSCs.

Flow cytometry analysis was performed to examine the enrichment of BCSCs in control and NuMA1 KO cells. Briefly, cells were incubated with Aldefluor reagent (Stem Cell Technologies, 01700) in presence/absence of DEAB for 30 minutes according to manufacturer’s instructions. CD29-FITC (Biolegend, 102206) and CD61-PE antibodies (Biolegend, 104308) were incubated with cells for 20 min at 4 °C followed by flow cytometry analysis (FACS Canto, BD Biosciences). Data was analysed using Flow Jo software. For isolation of BCSCs, control and NuMA1 KO cells of BF3M was trypsinized and counted in PBS. Cells were then incubated with CD29-FITC (Biolegend, 102206), CD61-PE (Bio-legend, 104308) antibodies for 30 min for isolation of CD29^hi^ CD61^+^ BCSCs. BCSCs were utilized for the subsequent experiments after isolation and not cultured for more than 4 days [25, 36].

### Migration assay

Scratch assay was performed to evaluate the migration potential of BCSCs and bulk cancer cells. Cells were plated in 24-well plate at a density of 1 × 10^5^ cells/well. A scratch was made in the middle of the well with a sterile pipette tip followed by PBS wash to remove the unattached cells. Images were captured at different time intervals to analyse the migration of cells using ImageJ as described previously [30, 36]. For Boyden chamber assay, cells were placed on upper chamber of inserts with basal media and chemoattractant (10% FBS) added to the lower chamber as described previously [37].

### Invasion assay

Inserts were placed in a 24-well low evaporation companion plate and coated with growth factor reduced Matrigel (Corning, 354230) for 1 h at 37 °C. In upper chamber, cells at a density of 30,000 cells/well were added with basal medium and the lower chamber was filled with 10% FBS as chemoattractant. Cells were incubated for 16 h at 37 °C and the cotton swabs were used to remove the cells on top of the membrane. Cells invaded to the lower chamber were quantified by fixing with 4% PFA followed by staining with crystal violet [36, 38].

### Cell viability

MTT assay was performed to examine the effect of inhibitor, SMI-4a on BCSCs and bulk cells as described previously [5, 30]. Briefly, cells were plated in 96-well plate at a density of 10 × 10^3^ in complete medium. After 24 h, inhibitors were added with varying concentrations from 0.01 to 100μM for 48h. Separately, BF3M cells were also treated with Lys-05, SMI-4a and combination (sum of the two drugs doses showed on the graphs) of these drugs at a concentration of 0.01–100μM for 48h. Absorbance was measured at 570nm after addition of MTT reagent for 90min. The IC_50_ values for the inhibitors against cells were calculated using Graphpad prism software [5].

### Human tissue microarray (TMA)

We purchased two human breast cancer tissue microarrays from US Biomax, (#BC081116e) to analyse the expression levels of NuMA1 and PIM1. Each TMA consist of 110 cores of paraffin embedded tissue samples. Among them, 100 samples from breast carcinoma and 10 from adjacent normal breast tissue. These tissue sections were utilized for IHC analysis and followed by immunostaining with NuMA1 and PIM1 primary antibodies [39].

### Statistical analysis

Results are represented as mean ± SEM of at least three or more individual experiments. All the results and IC_50_ were calculated from typical experiments reproduced at least thrice with similar results. Statistical analysis was performed between control and test groups using one/two –way ANOVA followed by Tukey’s post-hoc analysis or Sidak’s multiple comparison test and one sample two tailed *t* test with p values ≤0.05 considered significant.

## Results

### High NuMA1 expression correlates with poor survival in TNBC patients and its deletion in BF3M cells reduces tumorigenicity.

**Fig. 1.**
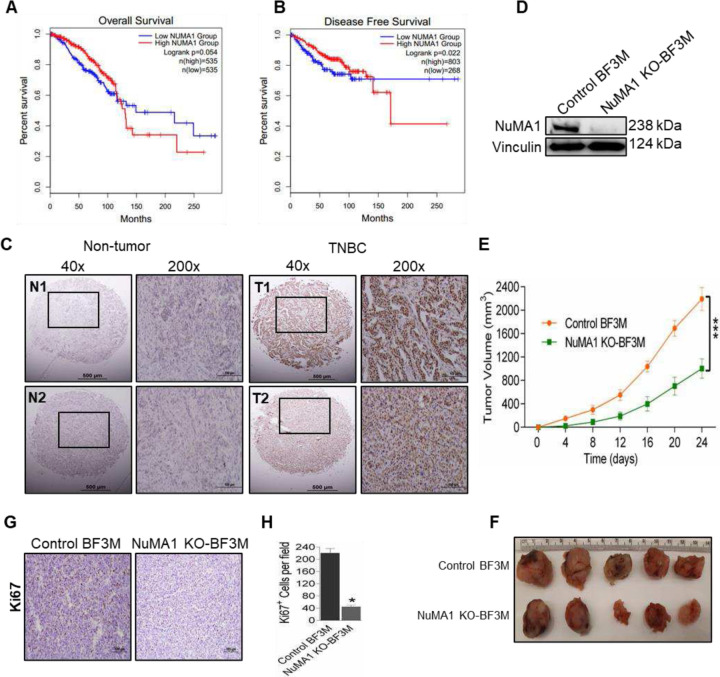
Upregulation of NuMA1 associates with poor survival in TNBC patients and ablation of NuMA1 inhibits tumorigenicity (**A**) Overall survival and (**B**) disease free survival of breast cancer patients retrieved from TCGA data using GEPIA2 database. (**C**) Representative micrographs showing IHC staining of NuMA1 in TNBC from human breast cancer tissue microarray (n=110). Right side images are magnified areas from left side image indicated by square box. Scalebar represents 500μm (left); 100μm (right). (**D**) Immunoblots showing CRISPR mediated knockout of NuMA1 expression in BF3M cells. Vinculin is used as a loading control. n = three independent experiments. (**E**) Tumor volumes of control BF3M and NuMA1 KO-BF3M cells. Mean ± SEM; Sidak’s multiple comparison test ****p < 0.0001. n = 5 mice per group. (**F**) Tumor growths of mice transplanted with control BF3M and NuMA1 KO-BF3M cells into mammary fat pad. **(G, H**) Images showing Ki67 staining and its quantification in tumor tissues of control BF3M and NuMA1 KO-BF3M cells. Mean ± SEM; one-sample two-tailed *t* test *p < 0.05. n = 5 mice per group.

To evaluate the NuMA1 expression with the survival of breast cancer patients, we analysed TCGA data using GEPIA2 online software. High NuMA1 expression was significantly concomitant with decreased overall survival (Fig. 1A) and disease-free survival (Fig. 1B) of breast cancer patients. Next, we examined NuMA1 expression in human breast cancer tissue to determine its expression levels in TNBC subtype. NuMA1 expression was elevated in TNBC human breast cancer samples as compared to normal breast tissue (Fig. 1C), suggesting that NuMA1 expression was elevated in the TNBC subtype.

To identify the potential role for NuMA1 in the regulation of TNBC subtype, we performed CRISPR mediated knock out of NuMA1 in mouse breast cancer cells, BF3M which was derived from Brca1^F/F^ P53^F/F^ K14-Cre representing basal subtype of breast cancer[32–35]. Efficient deletion of NuMA1 is confirmed in the pooled KO cells (Fig. 1D) and mouse breast tumor tissues (Fig. S1A) derived from NuMA1 KO-BF3M cells. BF3M cells were then transplanted into the mammary fat pad of syngeneic FVB female mice to evaluate the effect of NuMA1 deletion on mammary tumor growth in vivo. NuMA1 KO-BF3M cells exhibited significantly reduced growth in the recipient mice compared to control BF3M cells at 24 days after transplantation, which was confirmed by the harvested tumors at the end points (Fig. 1E, 1F). We then examined the proliferative rate of these tumors by immune staining of tumor sections with Ki67. Tumors derived from NuMA1 KO-BF3M cells had significantly reduced Ki67 positive cells compared to those from control BF3M cells. (Fig. 1G, 1H), suggesting decreased proliferation of these tumor cells upon NuMA1 deletion. Together, these results suggest that NuMA1 plays a role in the mammary tumor cell growth in TNBC subtype.

### NuMA1 ablation reduces BCSCs and characterization of BCSCs and bulk cells sorted from BF3M cells.

**Fig. 2.**
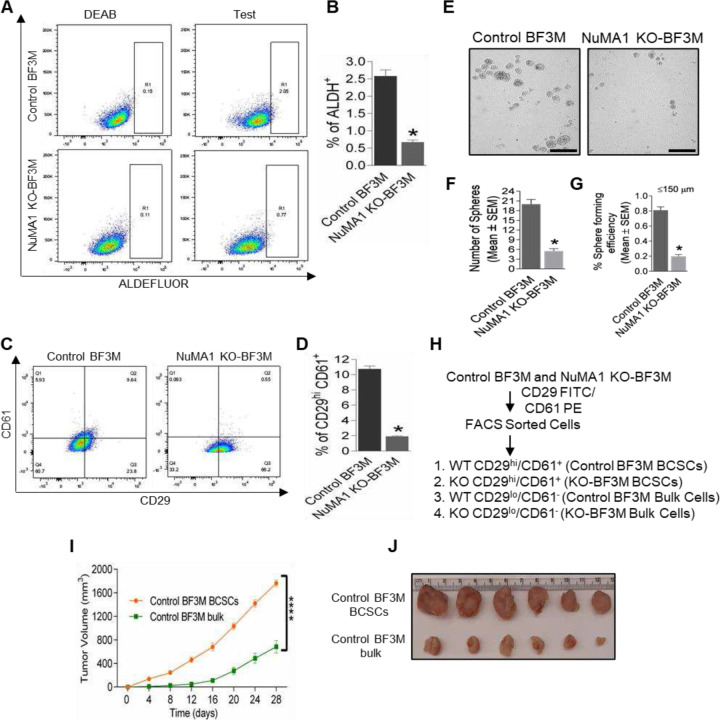
NuMA1 deletion decreases BCSCs and characterization of BCSCs and bulk cells sorted from BF3M cells. (**A, B**) Dot plots showing the gating of ALDH^+^ BCSCs in control BF3M and NuMA1 KO-BF3M cells relative to respective negative control (+DEAB) and its quantification (right panel). Mean ± SEM; one-sample two-tailed *t* test *p < 0.05. n = three independent experiments. (**C, D**) Dot plots depicting the CD29^hi^ CD61^+^ BCSCs in control BF3M and NuMA1 KO-BF3M cells. Quantified results significantly showed a smaller number of BCSCs in NuMA1 KO-BF3M cells. (**E, F, G**) Images are showing mammosphere formation, number of spheres and percent sphere forming efficiency of control BF3M and NuMA1 KO-BF3M cells. Mean ± SEM; one-sample two-tailed *t* test *p < 0.05. n = three independent experiments. (**H**) Flow chart showing isolation of BCSCs and bulk cells from BF3M cells using flow cytometry based on selection of CD29 and CD61 markers. (**I, J**) Orthotopic transplantation of control BF3M BCSCs and control BF3M bulk cells exhibited differential tumor growth and volume. Mean ± SEM; n = 5 mice per group; Sidak’s multiple comparison test ***p < 0.001; ****p < 0.0001.

To further study the role and mechanism of NuMA1, we analysed the effects of its deletion on sub-populations of tumor cells enriched in BCSCs using ALDH^+^ or CD29^hi^CD61^+^ as BCSC markers [25, 40]. Interestingly, we observed that NuMA1 KO-BF3M cells showed lower amounts of ALDH^+^ cells than control BF3M cells (Fig. 2A, 2B). Using CD29^hi^CD61^+^ as a marker and under similar gating criteria, we also found a lower fraction of BCSCs in NuMA1 KO-BF3M cells compared to those in control BF3M cells (Fig. 2C, 2D). Ablation of NuMA1 in BF3M cells showed a significant a smaller number of mammospheres (Fig. 2E, 2F) and reduced percent sphere forming efficiency (Fig. 2G) compared with control BF3M cells indicating that NuMA1 is not only modifying BCSCs number but also effecting its self-renewal ability, demonstrating the role of NuMA1 in BCSCs.

Previous studies have extensively characterized BCSCs in FF99 cells driven by PyMT showing their increased invasion and migration activity in vitro and tumorigenicity in vivo [25, 40]. We therefore examined potential differential functions of BCSCs vs bulk tumor cells by sorting them from BF3M cells using FACS based on CD29 and CD61 markers (Fig. 2H). We found that the BCSCs enriched by CD29^hi^CD61^+^ of BF3M cells showed increased migratory (Fig. S2A, S2B) and invasive (Fig. S2C, S2D) activities than bulk BF3M cells. Further, mammary fat pad transplantation assays revealed the increased tumor growth of BCSCs from BF3M cells relative to their respective bulk cells in the recipient mice in vivo, as measured by growth rate and tumor size at end points (Fig. 2I, 2J). These results suggest that like mammary tumors driven by BCSCs subpopulations depicted increased tumorigenic activity compared to the bulk tumor populations.

### Deletion of NuMA1 in BCSCs reduces metastasis of TNBC subtype.

**Fig. 3.**
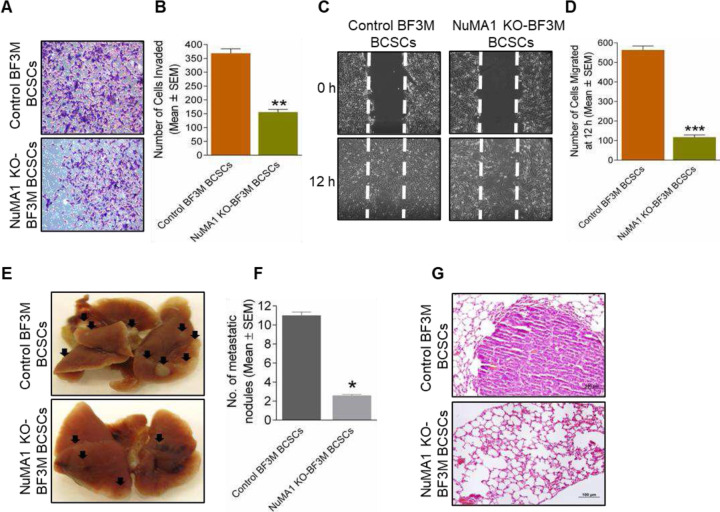
Deletion of NuMA1 in BCSCs reduces metastasis of TNBC subtype. (**A, B**) Representative images showing the number of invaded control BF3M BCSCs and NuMA1 KO-BF3M BCSCs and panel on the right shows quantification. (**C, D**) Representative wound healing assay results showing the rate of migration of NuMA1 KO-BF3M BCSCs significantly reduced as compared to control BF3M BCSCs at 12 hour time point and panel on the right shows quantification. Mean ± SEM; one-sample two-tailed *t* test **p < 0.01; ***p < 0.001; n = three independent experiments (**E, F**) Representative images show the formation number of metastatic lung nodules in mice injected with control BF3M BCSCs and NuMA1 KO-BF3M BCSCs via tail vein and bottom panel shows quantification. (**G**) H & E images of lungs depicting small size of metastatic foci in mice injected with NuMA1 KO-BF3M BCSCs. Mean ± SEM; one-sample two-tailed *t* test *p < 0.05. n = 5 mice per group.

Given the reduced subpopulations of BCSCs upon NuMA1 deletion, the above studies also suggested that NuMA1 plays a role in promoting mammary tumor cell activities in vitro and tumorigenicity in vivo through its positive function in maintaining BCSCs in TNBC subtype. To examine this hypothesis directly, we compared various activities of BCSCs upon NuMA1 deletion in BF3M cells. We found that CD29^hi^CD61^+^ subpopulation of BCSCs from NuMA1 KO-BF3M cells showed reduced activities in both invasion and migration assays relative to BCSCs from control BF3M cells (Figs. 3A–3D). We then examined the effects of ablating NuMA1 on the metastatic activities of BCSCs from BF3M cells. Consistent with the decreased migration and invasion in vitro, CD29^hi^CD61^+^ BCSCs from NuMA1 KO-BF3M cells showed significantly reduced number of lung metastasis nodules compared to that from control BF3M cells in tail vein injection experimental metastasis assays (Figs. 3E and 3F). Histological examination of lung sections revealed smaller size of lung nodules in the recipient mice injected with CD29^hi^CD61^+^ BCSCs from NuMA1 KO-BF3M cells (Fig. 3G), further supporting the reduced metastatic activity of BCSCs upon NuMA1 deletion in these cells. Together, these results demonstrate an important role for NuMA1 in BCSCs to promote metastasis in TNBC subtype.

### Regulation of PIM1 kinase in BCSCs migration and survival but not in bulk cells.

**Fig. 4.**
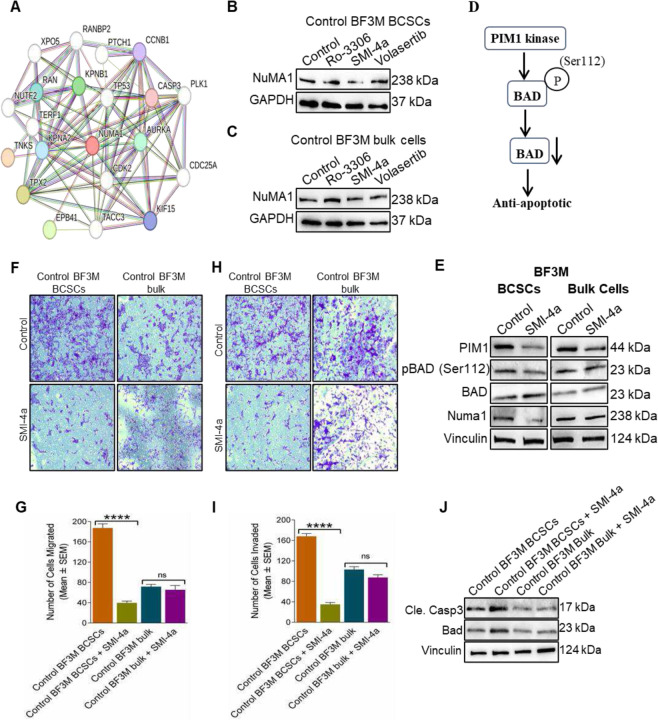
Activation of PIM1 kinase in BCSCs migration and survival but not in bulk cells. (**A**) Associated networks of NuMA1 using STRING database. (**B, C**) Immunoblots shows the expression of NuMA1 in BF3M BCSCs and bulk cells treated with cell cycle kinase inhibitors. n = three independent experiments. (**D**) Flow chart showing the PIM1 kinase phosphorylates BAD at serine 112 that leads to decreased BAD protein level and anti-apoptotic response. (**E**) Immunoblots showing the PIM1 kinase activity in BCSCs, but not in bulk cells, by reduced phosphorylation at ser 112 that led to decreased NuMA1 expression. (**F**) Representative images show the number of migrated control BF3M BCSCs and control BF3M bulk in presence/absence of SMI-4a, and (**G**) bottom panel shows quantification. (**H**) Representative images show the number of invaded control BF3M BCSCs and control BF3M bulk cells treated with or without SMI-4a, and (**I**) bottom panel shows quantification. Data are the Mean ± SEM of three independent experiments for both panels A and B; ns = no significance; Sidak’s multiple comparison test ****p < 0.0001. (**I**) Immunoblots showing the expression of cleaved caspase3, BAD and Vinculin in BF3M BCSCs and BF3M bulk cells treated with SMI-4a.

Although our results thus far indicate an important role for cell cycle protein, NuMA1 in the heterogenous populations of breast cancer subtypes examined, pharmacological inhibitors targeting NuMA1 are not yet available. For that reason, we aimed to identify cell cycle kinases that can regulate NuMA1. Thus, we analysed NuMA1 association networks using STRING database, and found that three major kinases in cell cycle regulation including CDK1 (CCNB1), PLK1 and PIM1, an interacting kinase of CDC25A (Fig. 4A). We then treated BCSCs with specific kinase inhibitors for these kinases including Ro-3306 (CDK1), SMI-4a (PIM1) and Volasertib (PLK1) and examined their effects on NuMA1. Interestingly, BCSCs sorted from BF3M (Fig. 4B) treated with SMI-4a, but not the other two kinases, depicted reduced expression of NuMA1 compared with control cells. In contrast, BF3M bulk cells treated with SMI-4a was not reduced NuMA1 expression (Fig. 4C). The flow chart is showing that PIM1 kinase activity, where PIM1 kinase phosphorylates the Bad at Ser112, thus reduces Bad protein expression and leads to anti-apoptotic response in cancer cells (Fig. 4D). PIM1 kinase inhibitor, SMI-4a treated BCSCs depicted reduced phosphorylation of Bad at Ser112, further it decreased the expression of NuMA1 as compared to untreated cells (Fig. 4E). In contrast, bulk cancer cells treated with SMI-4a could not reduce the phosphorylation of Bad at Ser112 as well as the downstream expression of NuMA1 (Fig. 4E), indicating that PIM1 kinase is specifically activating in BCSCs by regulating NuMA1 expression but not in bulk cells. We therefore tested the effect of PIM1 kinase inhibition in BCSCs and bulk tumor cells and found that treatment of cells with a PIM1 kinase inhibitor SMI-4a significantly decreased migration and invasive activities of BF3M BCSCs but had little effect on the corresponding bulk tumor cells (Figs. 4F–4I). Moreover, SMI-4a induced apoptosis of BF3M BCSCs, but not bulk BF3M tumor cells, as measured by apoptotic markers, cleaved caspase3 and Bad (Fig. 4J). These results suggest PIM1 kinase inhibition preferentially reduces NuMA1 levels in BCSCs, but not in bulk cells, to decrease cell migration and survival.

### Inhibition of PIM1 kinase in bulk cells increases autophagy (FIP200) through PIM1-MYC independent pathway.

**Fig. 5.**
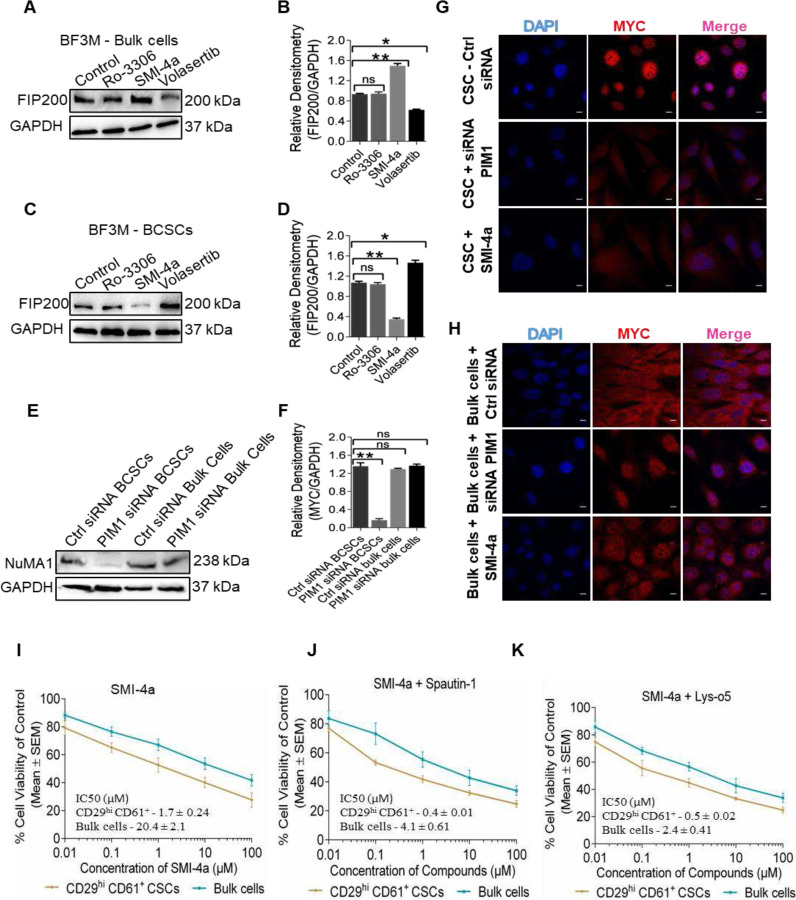
Inhibition of PIM1 kinase in bulk cells increases autophagy (FIP200) through PIM1-MYC independent pathway. (**A, B**) Immunoblots depicts expression of FIP200 in BF3M bulk cells treated with kinase inhibitors, Ro-3306, SMI-4a and volasertib. Right panel shows the quantification of relative densitometry of fig. A. Mean ± SEM; one-sample two-tailed *t* test *p < 0.05. n = three independent experiments. (**C, D**) BF3M BCSCs treated with kinase inhibitors and examined FIP200 protein expression using immunoblot. Right panel showing the quantification of fig. C. Mean ± SEM; one-sample two-tailed *t* test *p < 0.05. n = three independent experiments. (**E, F**) Immunoblots showing the expression of NuMA1 in BCSCs and bulk cells sorted from control and PIM1 siRNA BF3M cells. Right panel is quantification of fig. E. Mean ± SEM; one-sample two-tailed *t* test *p < 0.05. n = three independent experiments. (**G**) Confocal images showing the MYC nuclear accumulation in BF3M BCSCs in presence/absence of PIM1 siRNA and SMI-4a. (**H**) BF3M bulk cells with or without PIM1 siRNA and SMI-4a depicted MYC nuclear translocation. (**I-K**) MTT assay results represent cell viability of BF3M BCSCs and bulk cells treated with SMI-4a and in combination with autophagy inhibitors, spautin-1 and Lys-o5. Data represents the mean of three independent experiments. IC_50_ (μM) values showed in Mean ± SEM.

Previous studies reported that SMI-4a treatment induces autophagy by inhibiting AKT/mTOR pathway in various tissue specific cancer cells[41, 42]. To evaluate the role of autophagy in bulk cells, we treated sorted bulk cells using kinase inhibitors including Ro-3306 (CDK1), SMI-4a (PIM1) and Volasertib (PLK1) and observed their effects on FIP200 expression, an essential autophagy gene. BF3M bulk cells treated with SMI-4a, but not other kinase inhibitors showed a significant increased expression of FIP200 compared to control BF3M bulk cells (Figs. 5A and 5B). In contrast, BF3M BCSCs treated with SMI-4a showed reduced expression of FIP200 (Figs. 5C and 5D), indicating PIM1 kinase mediated regulation of FIP200 in BCSCs but not in bulk cells. It has been known that PIM1 kinase is important for MYC nuclear accumulation in breast and prostate cancer cells[15, 43]. Thus, we evaluated the role of MYC in BF3M BCSCs and bulk cells using siRNA mediated silencing of PIM1 (Fig. S3A). Interestingly, PIM1 siRNA BCSCs showed decreased NuMA1 expression as compared to control siRNA BCSCs but not in bulk cells, indicating role of MYC in BCSCs (Fig. 5E and 5F). Furthermore, nuclear accumulation of MYC in BCSCs was diminished upon silencing of PIM1 using siRNA and SMI-4a but not in bulk cells (Fig. 5G and 5H). Further, PIM1 kinase inhibition by SMI-4a and siRNA showed downregulation of FIP200 mRNA in BF3M BCSCs, not in bulk cells (Fig. S3B, S3C), indicating PIM1 kinase mediated MYC nuclear accumulation in BCSCs is important for its downstream transcriptional activation of FIP200. Consistent with these results, SMI-4a reduced viability of CD29^hi^CD61^+^ BCSCs from BF3M cells to a greater extent than bulk BF3M bulk tumor cells in a dose dependent manner (Fig. 5I). SMI-4a induced autophagy in BF3M bulk cells showed highly sensitive to the autophagy inhibitors, spautin-1 (Fig. 5J) and Lys-o5 (Fig. 5K). These results suggest PIM1 kinase inhibition using SMI-4a showed increased autophagy in bulk cells, whereas in BCSCs preferentially reduces NuMA1 by inhibiting nuclear accumulation of MYC mediated transcriptional activation of autophagy gene, FIP200 in BCSCs.

### Combination therapy of PIM1 kinase and autophagy inhibition decrease breast cancer tumorigenicity and metastasis.

**Fig. 6.**
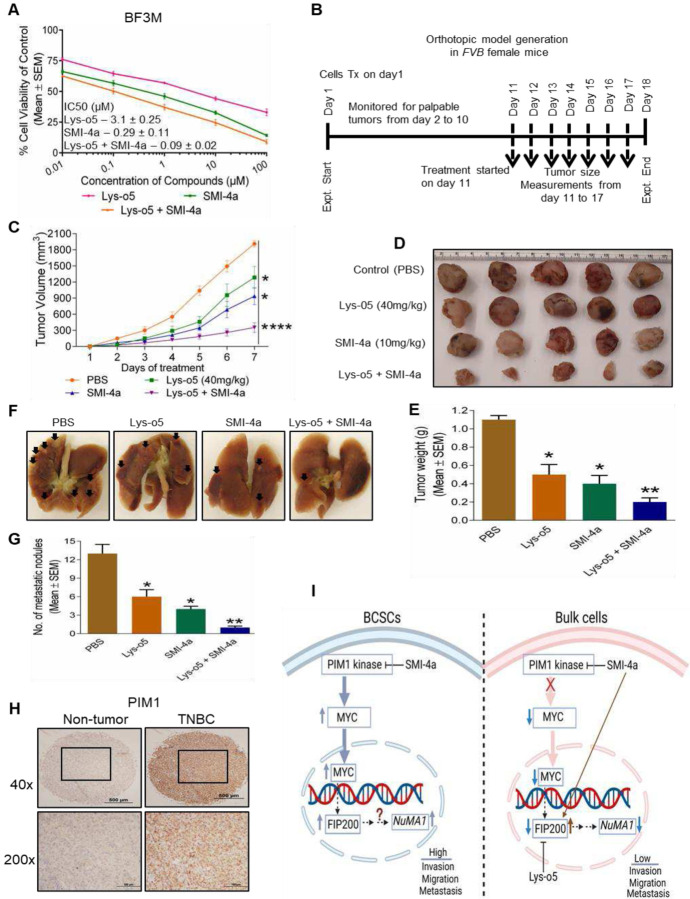
Combination therapy of PIM1 kinase and autophagy inhibition decrease breast cancer tumorigenicity and metastasis. (**A**) The percent cell viability of BF3M cells treated with individual treatments of Lys-o5, SMI-4a and combination (Lys-o5 + SMI-4a) (sum of the two drugs doses showed on the graphs). Data presented from the mean of three independent experiments. IC_50_ (μM) values displayed in Mean ± SEM. (**B**) Schema of experiments in panels E-I. On day 1, BF3M cells were transplanted into the mammary fat pad of FVB female. From next day, mice were monitored for palpable tumor formation. On day 11, mice were treated intraperitoneally with indicated drugs for 7 days. On day 18, tumors were harvested for tumor weight measurements. (**C**) Tumor growths of immunocompetent mice transplanted with BF3M cells and administered then with indicated drugs at specified doses for 7 days. (n=5 mice per group) Mean ± SEM; Sidak’s multiple comparison test ****p < 0.0001; n = 5 mice per group. (**D**) Tumor size of mice reduced over 7 days (started on day 11 after appearance of palpable tumor) with the combination treatment of (Lys-o5 + SMI-4a) than individual treatments. (**E**) Bar charts showing the weights of harvested tumors from mice treated with indicated drugs for 7 days. (**F**) Representative images of lung tissues are showing metastatic nodules derived from nude mice transplanted with BF3M cells and treated with indicated drugs. (**G**) Bar chart showing quantification of lung metastatic nodules from nude mice administered with PBS, Lys-o5, SMI-4a or Lys-o5 + SMI-4a. Mean ± SEM; one-sample two-tailed *t* test n=5 mice per group; *p < 0.05. **p < 0.01. (**H**) IHC staining of PIM1 expression in Non-tumor and TNBC breast cancer tissues depicted significantly higher PIM1 as compared to non-tumor tissues. Bottom images are magnified areas from top image indicated by square box. Scalebar represents 500μm (top); 100μm (bottom). (**I**) Scheme showing that high NuMA1 expression showed increased invasion, migration, and metastasis in BCSCs which was attenuated with the Inhibition of PIM1 kinase by SMI-4a. Lys-o5 showed high efficacy in bulk cells treated with SMI-4a mediated increased autophagy (FIP200).

Previous studies also showed a role for autophagy in promotion of tumorigenesis and metastasis of breast cancer, including affecting their BCSCs [25, 44]. Therefore, we tested a combination treatment strategy using SMI-4a in combination with an autophagy inhibitor Lys05. Both SMI-4a and autophagy inhibitors, Lys05 treatment reduced viability of BF3M cells in a dose dependent manner, and the combination of these two inhibitors reduced it to greater extent than either alone as evident by IC_50_ values (Fig. 6A). Next, we examined the effect of combination treatment of autophagy and PIM1 kinase inhibition on mammary tumorigenesis and metastasis in vivo. BF3M cells were transplanted into the mammary fat pad of FVB female mice on day 1. After palpable tumor formation, mice were treated intraperitoneally on day 11 with PBS as a control, SMI-4a (10 mg/kg), Lys05 (40 mg/kg), and combination of these two inhibitors for 7 days (Fig. 6B). Compared to control mice treated with PBS, SMI-4a and Lys05 treatments both inhibited tumor growth in vivo (Fig. 6C). Consistent with in vitro results, combination of these two inhibitors reduced tumor growth to a greater extent in vivo also. Moreover, we also found greater levels of inhibition of tumor growth by the combination of the inhibitors, as measured by the size and weight of the tumors harvested at the end of experiments (Fig. 6D, 6E). We also evaluated the effect of combination inhibitors on metastasis. The mice treated with individual drugs exhibited a significant number of reduced metastatic nodules which was further decreased in combination of drugs, Lys05 and SMI-4a (Fig. 6F, 6G), indicating that the combination treatment approach is much more effective in attenuating the metastatic potential of breast cancer. Lastly, the body weight of mice was not changed significantly with the treatments of various inhibitors as compared to mice treated with control PBS (Fig. S4A) indicating the minimal toxicity of the dose of inhibitors used in these experiments. Taken together, these results suggest that combination treatment of PIM1 kinase and autophagy inhibition is more effective as a potential therapy for TNBC.

Finally, we examined the expression levels of PIM1 in human breast cancer tissue to determine its expression levels in TNBC subtype. The PIM1 expression in TNBC human breast tissue samples exhibited higher expression as compared to normal breast tissues (Fig. 6H). Together, these results demonstrated that PIM1 expression is elevated in TNBC subtype, consistent with its functional roles to maintain NuMA1 levels to promote tumorigenesis and metastasis. PIM1 kinase regulates MYC nuclear accumulation in BCSCs for transcriptional activation autophagy gene, FIP200 that mediated the expression of NuMA1. In contrast, bulk cells could not regulate NuMA1 expression by activation of PIM1 kinase and MYC nuclear translocation. PIM1 kinase inhibition by SMI-4a led to reduction of NuMA1 expression by PIM1-MYC pathway, but not in bulk cells instead it led to increased autophagy levels, as measured by FIP200 expression (Fig. 6I). Therefore, use of combination approach SMI-4a and Lys-o5 inhibitors against BCSCs and bulk cells, respectively are effective therapy for TNBC patients.

## Discussion

Breast cancer is recognized as a complex disease due to the presence of heterogeneity characterized by assortment in genomic alterations, treatment responses, gene expressions and metastatic behaviour [45]. In addition, breast cancer has been classified into various subtypes such as Luminal, HER2^+^, Basal and Normal-like breast cancer based on hormonal receptor expression and molecular complexity [46]. The available endocrine hormonal therapy is effective against ER^+^ or PR^+^ tumors while HER2-targeted therapies are beneficial for HER2^+^ breast cancer patients [47]. TNBC is more aggressive with absence of hormone receptors and few specific targeted therapies, chemo and radiotherapies has potential limitations on advanced breast cancer [48]. Overall, the high degree of heterogeneity (intra-tumor and inter-tumor) and variations in treatment responses of TNBC presents significant challenges for effective treatments [2, 45]. Thereby, utilization of combination therapy targeting heterogenous populations would be better treatment approach for TNBC.

Thus, to uncover an effective combination approach against TNBC subtype, we examined the role of a cell division gene, NuMA1 in TNBC cells, BF3M. We performed CRISPR mediated deletion of NuMA1 in BF3M cells (Fig. 1D). NuMA1 has been shown to have multiple functions in cell division such as interaction with microtubules, maintenance of mitotic spindles and regulation of symmetric as well as asymmetric cell divisions [9, 12]. Moreover, upregulation of NuMA1 has been observed in breast cancer and epithelial ovarian cancer [7, 49, 50]. However, the role of NuMA1 in TNBC is not known. We evaluated the role of NuMA1 on mammary tumorigenesis of distinct TNBC subtype (Figs 1E–1H) and demonstrated a pro-tumorigenic role of NuMA1 in TNBC. The involvement of NuMA1 in BCSCs has not been addressed in prior studies and surprisingly, ablation of NuMA1 in TNBC subtype leads to reduction of BCSCs, CD29^hi^CD61^+^ and ALDH^+^, demonstrating that NuMA1 is important for the maintenance of BCSC properties (Fig. 2A–2D).

Functional differences and differential properties exist between the CD29^hi^CD61^+^ and ALDH^+^ BCSCs derived from the same tumor [25, 51]. In our study, deletion of NuMA1 in BF3M cells led to reduced populations of these two BCSCs, suggesting a role for NuMA1 in BCSCs marked by either CD29^hi^CD61^+^ or ALDH^+^ BCSCs. This observation led us to isolate and study the role of NuMA1 in a single BCSC instead of two BCSC populations from TNBC subtype. Previously, our lab characterized CD29^hi^CD61^+^, ALDH^+^ BCSCs from FF99 cells [25] and here for the first time, we characterized CD29^hi^CD61^+^ BCSCs vs bulk cells from BF3M using in vitro (Figs S2A–S2B) and in vivo assays (Figs. 2I–2J). These putative surface markers will be utilized in future studies for in depth BCSCs related mechanisms. BCSCs have been known to initiate metastasis [36] which was significantly reduced upon deletion of NuMA1 in TNBC subtype, demonstrating that NuMA1 plays a pro- metastatic role (Fig. 3E–3G).

Unlike to our findings that PIM1 kinase regulates NuMA1 expression, it has been reported that PIM1 kinase interacts with NuMA1 in HeLa cells that had been inhibited during mitosis with nocodazole [52]. Surprisingly, PIM1 inhibitor (SMI-4a) treated BCSCs showed a significant reduction in migration and invasion activities as compared to bulk cells, indicating a specific role of PIM1 kinase in BCSCs. Our results were in accordance with a previous study which showed that stem cell-like characteristics were diminished upon deletion of PIM1 in breast cancer cells [53]. We showed that inhibition of PIM1 kinase using SMI-4a in BCSCs induced increased apoptosis markers expression such as Bad and Cleaved Caspase3 than bulk cells (Fig. 4J). Previously, it was not well-known that PIM1 kinase inhibition mediated apoptosis is specific to BCSCs. Additionally, recent reports indicated that PIM1 inhibition induced chemo-sensitization to drugs such as cisplatin and docetaxel in various tissue specific cancers [54], revealing that combinatorial treatment of PIM1 inhibition along with autophagy inhibitor might be an effective therapeutic approach against cancer.

For the first time, we observed that differential regulation of PIM1 kinase in BCSCs and bulk cells of TNBC subtype. Increasing evidence suggests that autophagy inhibition alone or in combination with other targeted therapies showed potential strategy for cancer treatments. Accordingly, in our study, the combinatorial treatment of PIM1 kinase and autophagy inhibition resulted in a significant reduction of mammary tumorigenesis (Fig. 6D, 6E) and metastasis (Fig. 6F, 6G), suggesting that this combination approach might have a strong clinical implication in TNBC subtype.

## Conclusions

In this study, we demonstrated a role for NuMA1 in the regulation of tumor phenotypes, mammary tumorigenesis, and enrichment of BCSCs. Moreover, PIM1 kinase preferentially regulated NuMA1 expression in BCSCs, and combinatorial treatment of PIM1 kinase inhibitor and autophagy inhibitor attenuated breast tumor progression and metastasis, suggesting a potentially new therapeutic approach for TNBC subtype.

## Supplementary Material

1

## Data Availability

The datasets used during the current study are available from the corresponding author on reasonable request.
