# Supplementary material for: Role of NuMA1 in breast cancer stem cells with implications for combination therapy of PIM1 and autophagy inhibition in triple negative breast cancer
Source: Res Sq. 2024 Apr 1:rs.3.rs-3953289. Preprint. [Version 1] doi: 10.21203/rs.3.rs-3953289/v1 (PMC11030541; doi:10.21203/rs.3.rs-3953289/v1)

Fig. S1. Deletion of NuMA1 inhibits tumorigenicity. (**A**) IHC of NuMA1 expression in tumor tissues derived from orthotopic transplantation of control BF3M and NuMA1 KO-BF3M cells. n = 5 mice per group.

Fig. S2. Characterization of BCSCs and bulk cells sorted from BF3M cells. (A) Wound healing images are showing that migration of control BF3M BCSCs and control BF3M bulk cells at 0 and 12 h. time point. **(B)** Bar charts show quantified results of fig. (A). Mean ± SEM;

one-sample two-tailed *t* test $^*p < 0.05$. n = three independent experiments. **(C, D)** Invasion images depicted the number of invaded control BF3M BCSCs and control BF3M bulk cells and its quantification (right panel). Mean ± SEM; one-sample two-tailed t test $^*p < 0.05$.

Fig. S3. Silencing of PIM1 reduces FIP200 expression in BCSCs. (A) Immunoblots showing expression of PIM1 in BF3M cells silenced using PIM1 siRNA. **(B)** FIP200 mRNA expression in presence/absence of PIM1 siRNA and SMI-4a in BCSCs. Mean ± SEM; one-sample two-tailed t test $^*p < 0.05$. **(C)** mRNA expression of FIP200 in BF3M bulk cells treated with or without SMI-4a and PIM1 siRNA. Mean ± SEM; one-sample two-tailed t test $^*p < 0.05$.

Fig. S4. Combination treatment of Lys-o5 and SMI-4a for TNBC. (A) The body weight of the mice not changed with the treatment of PBS, Lys-o5, SMI-4a and in combination of Lys-o5 and SMI-4a. Mean ± SEM.

Figure 1 Supplemental

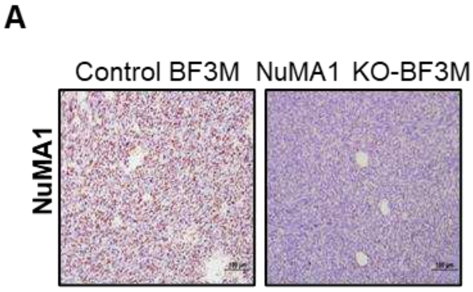

Figure 2 Supplemental

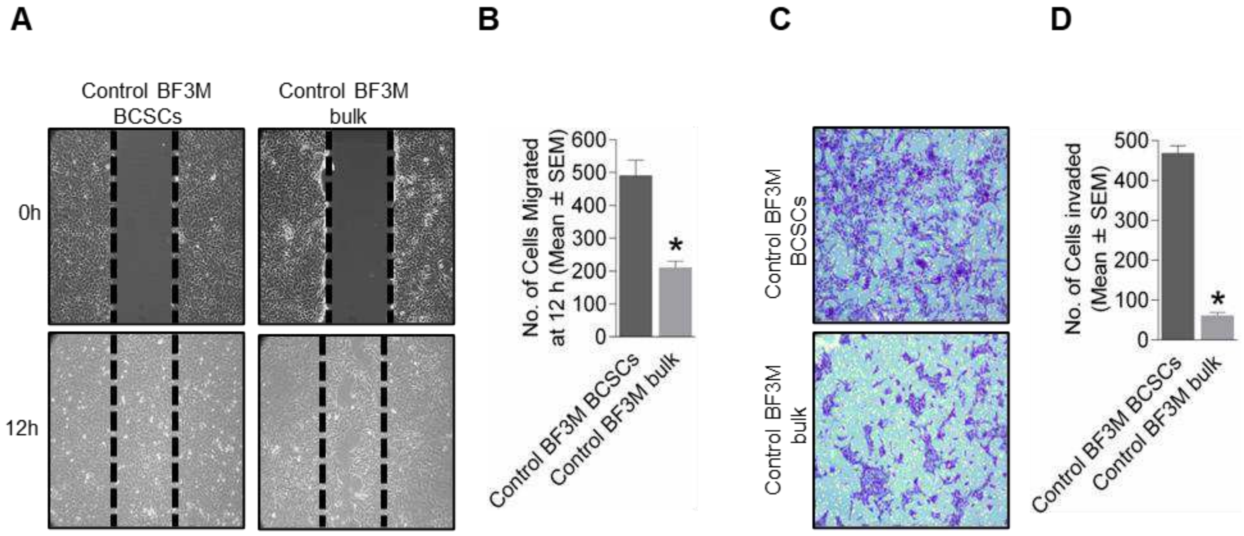

Figure 3 Supplemental

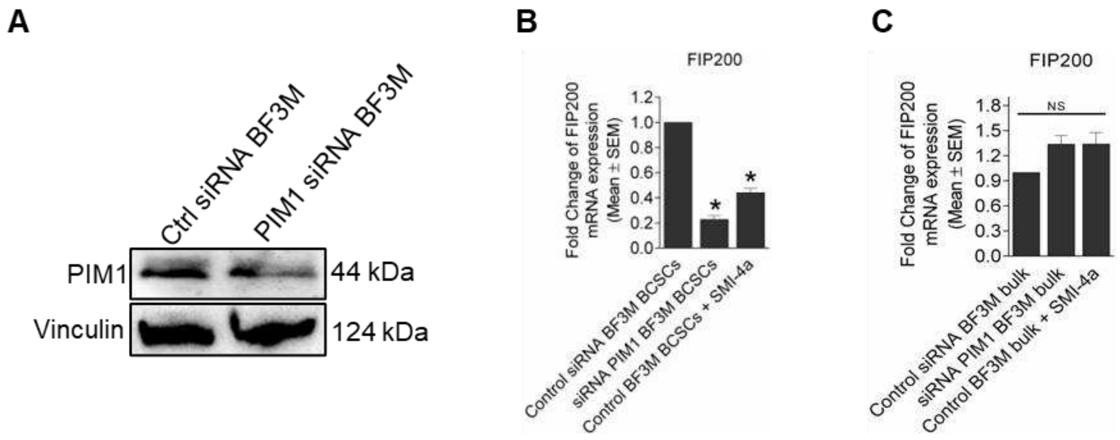

Figure 4 Supplemental

A

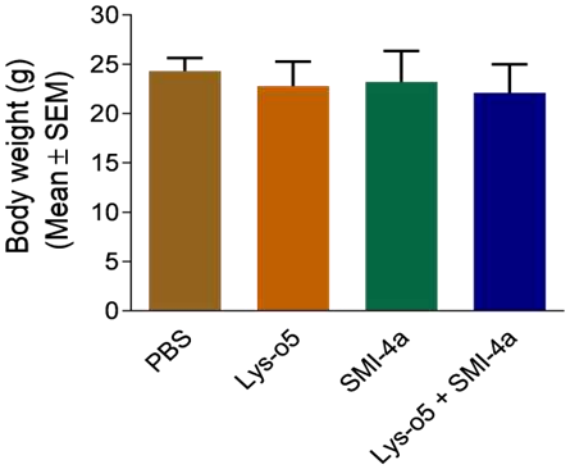

Supplement: 1 [file NIHPPrs3953289V1-supplement-1.pdf]
